# Multiomics integrative analysis for gene signatures and prognostic values of m^6^A regulators in pancreatic adenocarcinoma: a retrospective study in The Cancer Genome Atlas project

**DOI:** 10.18632/aging.103942

**Published:** 2020-10-20

**Authors:** Wenzhe Gao, Liuyang Cheng, Shuhan He, Wei Li, Chengyu Zhou, Bixia Zhou, Jiamiao Liu, Jiahao Xu, Xiao Yu, Hongwei Zhu

**Affiliations:** 1 Department of Hepatopancreatobiliary Surgery, The Third Xiangya Hospital, Central South University, Changsha 410013, Hunan Province, China; 2 Medical College of Xiangya, Central South University, Changsha 410013, Hunan Province, China; 3 Department of Gastroenterology, The Third Xiangya Hospital, Central South University, Changsha 410013, Hunan Province, China

**Keywords:** N6-methyladenosine, pancreatic adenocarcinoma, IGF2BP2, TCGA, ICGC

## Abstract

N6-methyladenosine(m^6^A) is the most abundant post-transcriptional RNA modification in eukaryotes. However, little is known about its role in pancreatic adenocarcinoma (PAAD). The aim of our study was to identify gene signatures and prognostic values of m^6^A regulators in PAAD. Patients from 3 different datasets with complete genomic and transcriptomic sequencing data were enrolled. Survival analysis for different gene alterations was performed using log-rank tests and Cox regression model. The association between alteration of m^6^A regulators and clinicopathological characteristics was examined using chi-square test. Results showed a high frequency of copy number alterations (CNAs) of m^6^A regulatory genes in PAAD patients, but somatic mutations were rarely happened. CNAs and mutations of m^6^A regulatory genes was associated with patient’s gender, pathologic stage and resected tumor size. Patients with “gain of function” for m^6^A “reader” genes combined with copy number loss of “writers” or “erasers” had worse overall survival (OS) compared with other patterns. Moreover, copy number gain of m^6^A “reader” gene *insulin growth factor 2 binding protein 2* (*IGF2BP2)* was an independent risk factor for OS (HR = 2.392, 95%CI: 1.392-4.112, p<0.001) and disease-free survival (DFS) (HR = 2.400, 95%CI: 1.236-4.659, p=0.010). Gene Set Enrichment Analysis (GSEA) indicated that *IGF2BP2* was correlated with multiple biological processes associated with cancer, of which the most significant processes were relevant to cancer cell cycle, cell immortalization and tumor immunity. To sum up, a significant relationship was found between m^6^A genomic alterations and worse clinical outcomes. These innovative findings are expected to guide further research on the mechanism of m^6^A in PAAD.

## INTRODUCTION

Pancreatic cancer (PC) is now the 9^th^ common malignancy worldwide and the most lethal cancer among all kinds of malignant diseases, the 5-year survival rate of which is only 9% in the United States. It is estimated that 57,600 people will develop PC in 2020 and 47,050 will die from it in America [1]. Pancreatic adenocarcinoma (PAAD) represents more than 95% of patients of all PCs [2], its unknown cause, difficulty of early diagnosis and rapid progress make it an extremely hard nut to crack for oncology researchers around the world. Resection is currently the only way to cure PAAD. However, clinical experience has shown that tumor invasion or distant metastasis occurred in most patients by the time of consultation and indication for surgery had been lost [3]. For these advanced patients, current adjuvant and neoadjuvant chemotherapy and radiotherapy could not show ideal improvement for prognosis. Thus, exploring the molecular mechanism underlying the pathogenesis and finding targets with prognostic and therapeutic potential became the most critical work to break through the diagnosis and treatment of PAAD. Encouragingly, the past 5 years have witnessed the rapid development of molecular subtyping of PAAD. Bailey P and his colleagues firstly identified 32 recurrently mutated genes and 10 aggregated pathways to define 4 molecular subtypes for PAAD: (1) squamous; (2) pancreatic progenitor; (3) immunogenic; and (4) aberrantly differentiated endocrine exocrine (ADEX) [4]. This was the first research establishing an association between molecular features and histopathological subtype of PAAD. The continuous addition and development of molecular taxonomies since then have accelerated the development of individualized therapeutic methods and accurate prediction of patients’ survival [5]. However, until today, new and enormous molecular features are still needed to further understand the relationship between molecular pathological alterations and clinical manifestations of patients.

Epigenetics refer to changes in gene expression that are stable during cell division, and sometimes stable between generations, without changing in DNA sequence. Epigenetic changes related to DNA and histone, like methylation and acetylation, have been widely studied in PAAD progression over the past decades [6]. In recent years, the attention of scientists has gradually shifted to the study of RNA epigenetics. So far, more than one hundred kinds of chemically modified nucleotides have been found in different types of RNAs, of which the earliest discovered one was N6-methyladenosine(m^6^A) [7]. This reversible RNA modification is very abundant in eukaryotes. So far, over 7600 mRNAs of protein coding genes and hundreds of non-coding RNAs have been confirmed to have the presence of m^6^A [8], with approximately 3-5 adenylate residues on average appearing in each mRNA [9]. These m^6^A sites are mainly located in the 3’UTR region of mRNA near the stop codons and long internal exons [10]. M^6^A modification is mainly driven by three kinds of regulatory proteins (enzymes): methyltransferase complex (“Writers”), demethylases (“Erasers”) and reader proteins (“Readers”). “Writers” serve to mediate the methylation modification process of adenylate residues on RNAs. In contrast, “Erasers” mediate the demethylation process of RNA, reversing chemical response catalyzed by the “Writer complex”. “Readers” are responsible for "reading" the information of RNA methylations and are involved in downstream RNA processing, including exportation of RNAs from nucleus, post-transcriptional splicing, mRNA translation, RNA decay and many other processes related to RNA stability [11].

M^6^A dysregulation is involved in diverse pathophysiological processes, the most significant one of which is cancer. The review published by Chen X [12] and his colleagues in 2019 summarized the relationship between m^6^A and the pathogenesis of various malignancies. They concluded that dysregulated m^6^A modification could affect tumor proliferation, differentiation, tumorigenesis, invasion and metastasis by regulating mRNAs of proto oncogenes and tumor suppressor genes in post-transcriptional level. Moreover, many m^6^A regulatory proteins played a double-sided role in promoting or suppressing cancer in different tumors, indicating that meticulous and complex m^6^A regulatory mechanisms existed in neoplastic diseases. There has been some research focusing on the relationship between m^6^A and PAAD. Zhang J et al [13] found that the “writer” protein METTL3 could promote PAAD by enhanced m^6^A modification of microRNA-25 (miR-25), leading to the excessively maturation of miR-25 and further activation of oncogenic AKT-p70S6K signaling. Another research from Tang B et al [14] demonstrated that m^6^A “eraser” ALKBH5 was downregulated in gemcitabine-resistant xenograft models and its overexpression sensitized PAAD cells to chemotherapy. This mechanism might be related to the activation of Wnt signaling pathway. Since little has been known about the role of other m^6^A regulatory genes in the pathogenesis of PAAD so far, this study aimed to explore gene signatures and prognostic values of all the currently recognized m^6^A regulators by analysis of genomic and transcriptomic data in The Cancer Genome Atlas (TCGA) project and other available datasets using bioinformatic methods.

## RESULTS

### Mutations and CNAs of m^6^A regulatory genes in TCGA_PAAD cohorts

**Figure 1:**
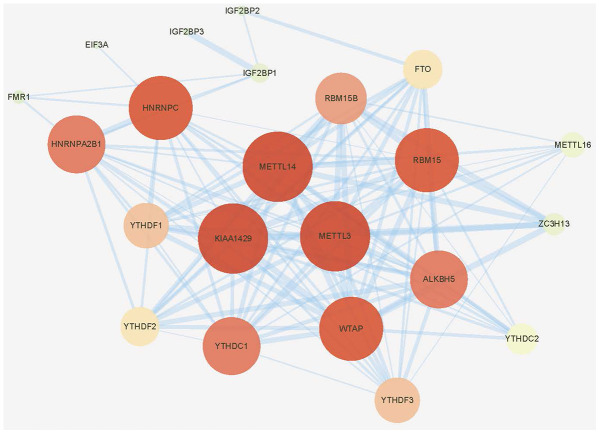
**Protein-protein interaction (PPI) network of the 24 included m^6^A regulators.**

M^6^A-related research has grown rapidly in recent years. To include all genes that have been experimentally confirmed to yield m^6^A regulating functions as much as possible, we first searched PubMed for all literatures relating to m^6^A. After carefully archiving, 2 reviews published in 2019 that comprehensively summarized all the m^6^A regulatory genes was adopted [15, 16]. Finally, twenty-four genes were included in subsequent studies by intersecting with the genome and transcriptome-sequencing gene set on TCGA, including 9 “writers”, 13 “readers” and 2 “erasers”. Protein-protein interaction (PPI) network analysis was then applied using “STRING” database (https://string-db.org/) and CytoScape software (National Resource for Network Biology, USA) to display the interaction between these m^6^A regulators. As shown in Figure 1, three “writer” genes, METTL3, METTL14 and KIAA1429 (also known as VIRMA) seemed to be the hub genes in this network. This result brought some preliminary hints for our next research.

**Figure 2:**
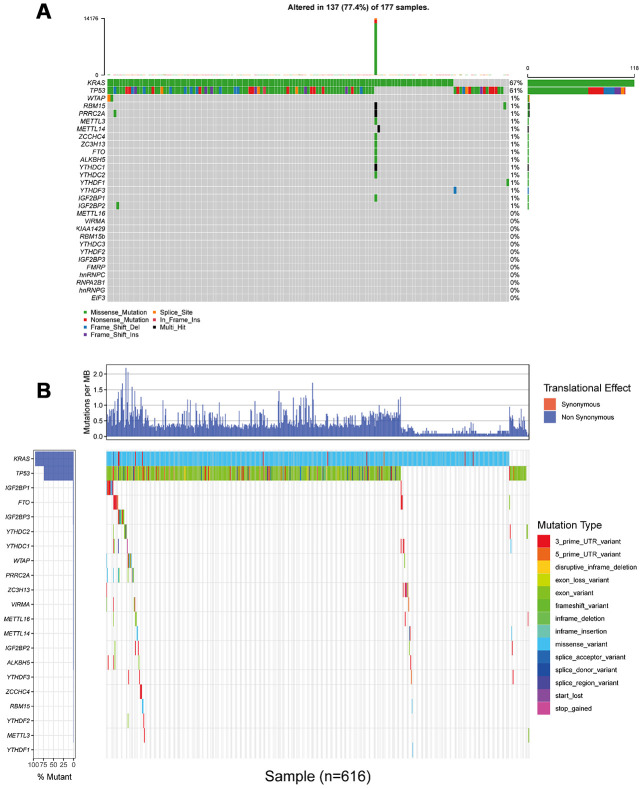
**Landscape of somatic mutation status of m^6^A regulators in PAAD.** (**A**) The mutation frequency of 24 m^6^A regulators in 177 patients from TCGA_PAAD cohort. Each column represented individual patients. The upper barplot showed tumor mutation burden (TMB). (**B**) The mutation frequency of 24 m^6^A regulators in 616 patients from ICGC_AU_PAAD and ICGC_CA_PAAD cohort. The upper barplot showed mutation events happeded in per 1 million bases. Due to the difference in the definition and naming of somatic mutations, it is unable to integrate (**A**) and (**B**) into the same waterfall chart.

To analyze somatic mutations of m^6^A regulatory genes, three cohort from different countries with complete genomic sequencing data and clinical information were included, they were TCGA_PAAD cohort, ICGC_AU_PAAD cohort and ICGC_CA_PAAD cohort, respectively (ICGC: international cancer genome consortium; TCGA: from America; AU: from Australia; CA: from Canada). All included genomic sequencing data were got from the same sequencing platform (Illumina HiSeq 2000, SAN DIEGO, USA). Among all 753 patients with complete sequencing data and clinical information, mutations of 24 m^6^A regulatory genes were found in 53 (7.04%) independent samples (Figure 2, Table 1, and Supplementary Table 1). On an individual level, most of these 53 cases had only one single nucleotide mutation of a certain m^6^A regulatory gene (Table 1 and Supplementary Table 1). The only one exception was from TCGA_PAAD cohort (ID: TCGA.IB.7651.01) who suffered a huge m^6^A mutation burden, with 21 non-synonymous mutations occurred in 11 m^6^A regulatory genes. From a genetic point of view, none of the included m^6^A genes had a mutation frequency of more than 1% in TCGA and ICGC database; compared with the mutation frequency of control genes *KRAS* and *TP53*. Thus, we had enough reason to believe that mutations of m^6^A regulatory genes were not a common event in PAAD patients (Figure 2). More specifically, a total of 6 patients had 6 mutation events in *WTAP*, made it the gene with the highest incidence of mutations in all 753 included patients; another 4 and 3 patients had 10 mutations in *IGF2BP1* and *YTHDC1*, respectively. *IGF2BP1* and *YTHDC1* became the genes with the highest number of mutation events.

**Table 1:** Mutations of m^6^A regulatory genes in 149 TCGA_PAAD patients.

**TCGA Sample ID**	**ALKBH5**	**EIF3A**	**FMR1**	**FTO**	**HNRNPC**	**IGF2BP1**	**IGF2BP2**	**METTL14**	**METTL3**	**RBM15**	**RBM15B**	**WTAP**	**YTHDC1**	**YTHDC2**	**YTHDF1**	**ZC3H13**	**ZCCHC4**
TCGA.IB.7644.01												C161Y					
TCGA.IB.7651.01	R327H	H204Y	R454H,A516D	R473W		G175D, P182S, A519T, Q563H			A191V	R717*, A185V			K565N, X374_splice, E224K, Q168H	G15V, V681I		H391Y	E505G, Y377C
TCGA.HZ.8002.01																K969I	
TCGA.HZ.8003.01					S209Y												
TCGA.F2.A44G.01								R298H, X23_splice									
TCGA.HV.A5A3.01							V559I										
TCGA.IB.A5SQ.01															R404C		
TCGA.IB.A5ST.01											R529W						
TCGA.IB.AAUS.01												K155E					
TCGA.2L.AAQE.01		T216M															
TCGA.LB.A9Q5.01		P1048L															
TCGA.2J.AABV.01									R471H								
TCGA.HZ.A9TJ.01												X49_splice					
TCGA.IB.AAUQ.01		A1012V															

**Figure 3:**
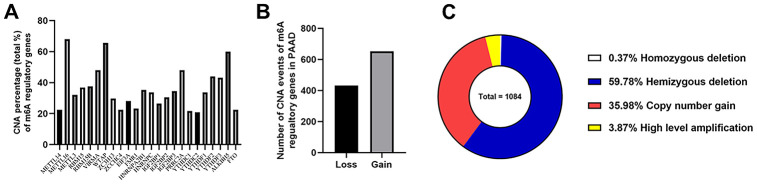
**Landscape of CNA events for m^6^A regulatory genes in PAAD.** (**A**) Percentage of PAAD samples with CNAs of the m^6^A regulators based on the data from TCGA. (**B**) Events of copy number gain or loss of m^6^A regulatory genes in ccRCC samples. (**C**) Proportion of four kinds of CNA vents.

Given that no copy number alteration (CNA) related data was provided in two datasets from ICGC database, TCGA_PAAD cohort became our only remaining data source for the next CNA analysis. In this cohort, 125 of these 149 cases (83.89%) had CNA events of m^6^A regulatory genes, which meant that CNAs occurred very frequently in PAAD patients. The “writer” gene *METTL16* had the highest frequency of CNA events (57.05%, 85/149), followed by *WTAP* and *ALKBH5*, which were “writer” and “eraser” genes, respectively (Figure 3A).

Then we evaluated CNA patterns in TCGA_PAAD cohort. The total number of CNA events was 1084 and most events led to the copy number loss of m^6^A genes (60.15%, 652/1084) (Figure 3B, 3C), which consisted of homozygous and hemizygous deletion, indicating the loss of two alleles, or one (or less than one) allele for a certain gene from genome in a diploid organism. In detail, hemizygous deletion of *METTL16* (82 cases) was the most frequent alteration in all kinds of CNAs of m^6^A regulatory genes (Table 2), followed by the hemizygous deletions of *WTAP* (79 cases) and *ALKBH5* (69 cases). The second most frequent CNA event was copy number gain (35.98%, 390/1084), which happened to *VIRMA* and *HNRNPA2B1* in 40 patients, tied for the first place, followed by the “reader” gene *IGF2BP3* (38 cases). To verify the reliability of this cohort, we also detected *KRAS* (all 149 cases with mutations and 39 cases with CNAs) and *TP53* (94 cases with mutations and 72 with CNAs) as control, which were also widely recognized by the published literatures.

**Table 2:** Different CNV patterns occur in TCGA_PAAD samples (n=125).

**Gene names**		**Homozygous deletion**	**Hemizygous deletion**	**Copy number gain**	**High level amplification**	**CNA sum**	**Diploid**
**Writers**	**METTL14**	0	25	3	0	28	97
	**METTL16**	1	82	2	0	85	40
	**METTL3**	0	17	22	1	40	85
	**RBM15**	0	35	11	0	46	79
	**RBM15B**	0	42	5	0	47	78
	**VIRMA**	0	10	40	10	60	65
	**WTAP**	0	79	3	0	82	43
	**ZC3H13**	0	13	24	0	37	88
	**ZCCHC4**	0	22	6	0	28	97
							
**Readers**	**EIF3A**	0	24	10	1	35	90
	**FMR1**	0	14	11	4	29	96
	**HNRNPA2B1**	0	2	40	2	44	81
	**HNRNPC**	0	18	22	2	42	83
	**IGF2BP1**	1	14	15	3	33	92
	**IGF2BP2**	0	12	24	2	38	87
	**IGF2BP3**	0	2	38	3	43	82
	**PRRC2A**	0	48	8	4	60	65
	**YTHDC1**	1	24	2	0	27	98
	**YTHDC2**	0	19	7	0	26	99
	**YTHDF1**	0	7	34	1	42	83
	**YTHDF2**	1	50	4	0	55	70
	**YTHDF3**	0	14	35	5	54	71
							
**Erasers**	**ALKBH5**	0	69	3	3	75	50
	**FTO**	0	6	21	1	28	97
**Controls**	**KRAS**	1	10	23	5	39	86
	**TP53**	1	69	2	0	72	53

### Alterations of m^6^A regulatory genes were associated with clinicopathological characteristics and survival of PAAD patients

The relationship between alterations (CNAs and/or mutations) of m^6^A regulatory genes and the clinicopathological characteristics of PAAD patients were analyzed. Results showed that alterations of m^6^A genes were significantly associated with the patients’ gender, tumor grade and the maximum diameter of resected tumor. More concretely, male patients had a higher frequency of mutations or CNAs than female patients; patients with larger tumor volumes and higher histopathological grade also tended to show increased alterations of m^6^A genes (Table 3).

**Table 3:** Clinical pathological parameters of PAAD patients with or without mutations/CNAs of m^6^A regulatory genes.

		With mutations and/or CNAs*	Without mutations and CNAs*	**p value**
**Age^#^**	≤65	68	11	p=0.463
	>65	63	7	
**Gender**	Male	74	6	p<0.001
	Female	57	12	
**Metastasis (M)**	M0	58	10	p=0.549
	M1	4	0	
	MX	69	8	
**Nodes (N)**	N0	35	4	p=0.671
	N1	95	14	
	NX	1	0	
**Primary tumor (T)**	T1	4	1	p=0.471
	T2	13	3	
	T3	111	13	
	T4	2	1	
	unknown	1	0	
**Maxium diameter of resected tumor**	≤3cm	43	8	p<0.001
	>3cm	77	8	
	unknown	11	2	
**Stage**	I	10	2	p=0.669
	IIA	22	2	
	IIB	91	13	
	III	2	1	
	IV	4	0	
**Histological grade^&^**	G1	13	7	p=0.049
	G2	77	8	
	G3	39	4	
	G4	1	0	
**Histological_diagnosis**	PDAC	117	15	p=0.244
	Colloid Carcinoma	1	1	
	Other Subtype	12	2	
**Primary site**	unknown	1	0	
	Body	11	0	p=0.107
	Head	102	15	
	Tail	11	0	
	Other	7	3	

*With mutations and/or CNAs: Cases have mutant or CNA or mutant+CNV, confirmed through TCGA database. Without mutant and CNV: Cases with neither mutant nor CNV, confirmed through TCGA database. ^#^The reason for grouping by age 65 was because 65 is the median age of this cohort. ^**&**^Histological grade indicating the degree of tumor differentiation, G1: well differentiated; G2: middle differentiated; G3: poorly differentiated; G4: undifferentiated. Ambiguous variables (Nx, Mx, N/A, discrepancy and Gx) were excluded from chi-square test or non-parametric test.

**Figure 4:**
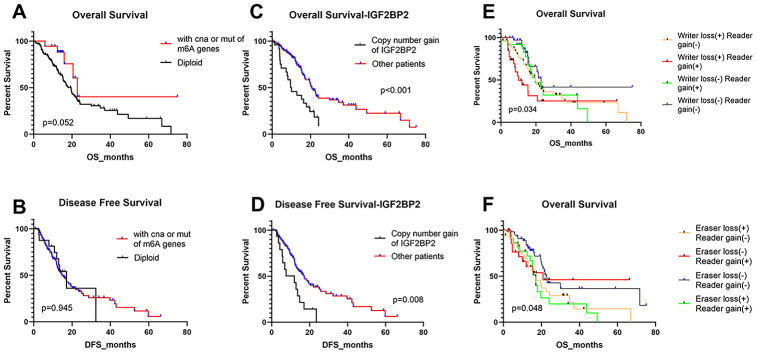
**Overall survival and disease-free survival of PAAD patients with different kinds of CNA and/or mutation patterns.** (**A**, **B**) OS and DFS for patients with any CNAs or mutations of m^6^A regulatory genes or with diploid genes. (**C**, **D**) OS and DFS for patients with copy number gain of IGF2BP2 or other patients except for them. (**E**) OS of PAAD patients with simultaneous alterations of writer genes and reader genes. (**F**) OS of PAAD patients with simultaneous alterations of eraser genes and reader genes.

The above results made us interested in the impact of these alterations on patients’ prognosis. Thus, we analyzed the effects of overall alterations of m^6^A regulatory genes and some certain high-frequency CNAs on patients’ overall survival (OS) and disease-free survival (DFS). However, results did not seem to be ideal. Individuals with or without mutations or CNAs of m^6^A regulatory genes didn’t have any significant correlation with OS and DFS (Figure 4A, 4B). Furthermore, separate analysis of the included 24 genes revealed that most single CNA events didn’t affect OS and DFS (Supplementary Figure 1, 2). The only exception is *IGF2BP2*, a m^6^A “reader” gene. Patients with copy number gain of *IGF2BP2* showed significantly poorer OS and DFS than other patients without this alteration (Figure 4C, 4D).

Then we divided the 24 m^6^A regulatory genes into 3 classes (writers, readers and erasers) based on their functions in m^6^A modification and detected whether the loss or gain of overall copy number for a class of m^6^A genes could influence patients’ OS and DFS. We found that copy number loss mainly happened to “writers” and “erasers”, yet “readers” mainly gained copy number. Therefore, patients were divided by whether “writer loss”, “eraser loss” or “reader gain” happened. It turned out that patients with “reader gain” combined with “writer loss”, and those with “reader gain” and “eraser loss” had worse OS (Figure 4E, 4F). Besides, no significant difference was found about DFS of these groups (Supplementary Figure 3A–3D). These results highlighted the potential importance of m^6^A reader genes on the prognosis of PAAD patients.

### CNAs of m^6^A regulatory genes were associated with mRNA and protein expression levels

**Figure 5:**
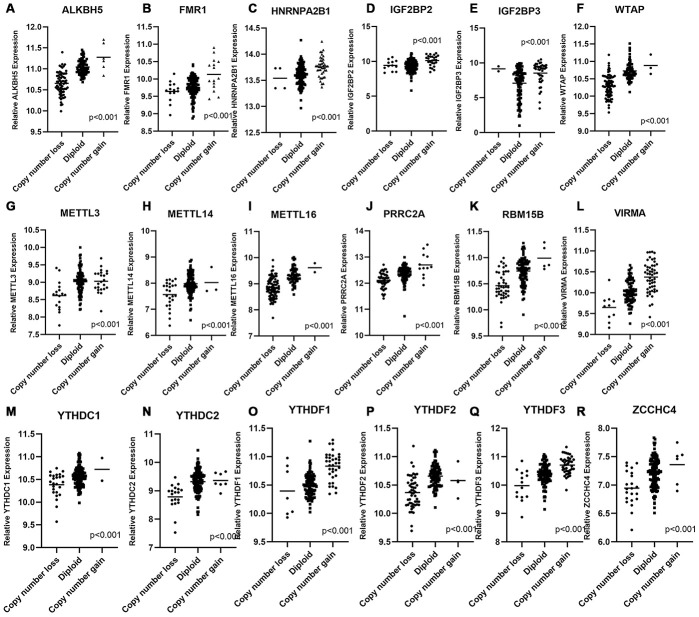
**Correlation between different CNA patterns and mRNA expression levels of m^6^A regulatory genes respectively.** (**A**–**R**) A total of 18 m^6^A regulatory genes showed significant correlation between their CNA patterns and mRNA expression. Only when p < 0.001 the gene was included in this figure, correlation plots of other m^6^A genes were listed in Supplementary Figure 4.

We wondered whether CNAs of m^6^A regulatory genes at the genomic level were related to their mRNA expression status. The results of correlation analysis showed that mRNA expression level was significantly associated with most of the CNA patterns in PAAD samples. In 21 of 24 detected genes, copy number gains and high-level amplifications were related to higher mRNA expression; while homozygous and hemizygous deletions resulted in a declined level of mRNA (Figure 5A–5R and Supplementary Figure 4A–4C). No significant relation was found between the CNAs of *FTO*, *RBM15* and *ZC3H13* and their mRNA expression level (Supplementary Figure 4D–4F).

Then we detected whether mRNAs of these 24 m^6^A regulatory genes are differentially expressed between PAAD patients and normal individuals. RNA-sequencing data from TCGA_PAAD, ICGC_AU_PAAD and ICGC_CA_PAAD dataset, and normal pancreas transcriptome data from Genotype-Tissue Expression (*GTEx*) database were enrolled, including 279 tumor samples and 328 normal pancreas samples. Unfortunately, none of the m^6^A regulatory genes met the conventional criterion of differential expression (log2FoldChange > 1.5, P < 0.05) (Supplementary Table 2). There were only 4 genes which showed statistically significant differential expression (P <0.05), among which the most differentially expressed one was *Insulin-like growth factor 2 mRNA binding protein 2* (*IGF2BP2*) (log2FoldChange = 0.341, P = 0.048). Heatmap showed that the expression of m^6^A -regulated genes cannot fully distinguish patients from normal individuals through clustering analysis, either (Supplementary Figure 5). However, some interesting hints drew our attention. First, the mRNA expression level of m^6^A regulatory genes were consistent with their copy number status; those genes that mostly showed copy number loss, like *METTL3*, *ALKBH5* and *YTHDF* family, all showed a tendency of lower expression compared with normal tissues; On the contrary, IGF2BP family, which mainly suffered copy number gain in PAAD patients, showed a tendency of mRNA over-expression in PAAD compared with normal pancreas. Besides, it was not difficult to notice from the heatmap that the most capable genes to distinguish tumors from normal tissues in Cluster Analysis were from IGF2BP protein family as well.

**Figure 6:**
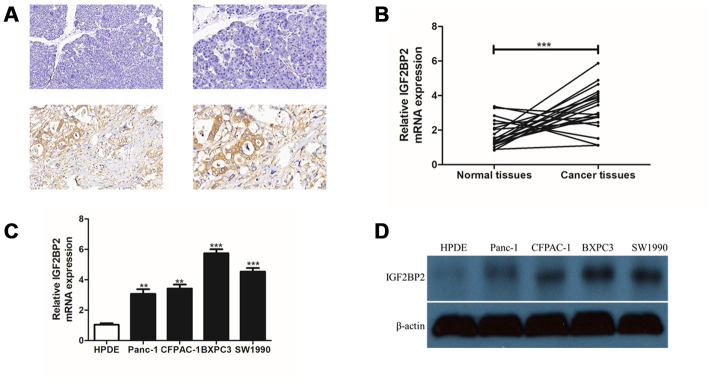
**Experimental validation of IGF2BP2 over-expression in PAAD tissue array and cell lines in vitro.** (**A**) Immunohistochemistry staining (IHC) for a pair of PAAD tissues and their adjacent normal tissues for IGF2BP2 protein. The upper part of the figure showed normal pancreas tissues and the lower part showed the corresponding PAAD tissue staining. The two pictures in left panel were magnified 200 times (200x), and the right panel, 400 times (400x). (**B**) Quantitative real-time PCR (qRT-PCR) for mRNA expression of *IGF2BP2* in 20 pairs of PAAD cells and their corresponding normal pancreatic cells. (**C**) qRT-PCR of *IGF2BP2* for 4 PAAD cell lines and one normal pancreatic ductal epithelial cell line (HPDE) in vitro. (**D**) Western Blotting (WB) showed the protein level of IGF2BP2 in 4 PAAD cell lines and HPDE cell line in vitro (**, P < 0.01; ***, P < 0.001).

Based on the above results, we had a strong interest in the expression status and mechanism of IGF2BP family in PAAD pathogenesis. IGF2BP family contained three members. As mentioned in our previous results, *IGF2BP1* had the highest mutation frequency, while *IGF2BP3* showed the highest CNA frequency; however, only *IGF2BP2* was associated with patient prognosis, and most likely there was a significant increase in its mRNA expression. Thus, IGF2BP2 was chosen as the candidate gene for further experimental detection. Immunohistochemistry staining for IGF2BP2 expression was firstly performed in 3 pairs of PAAD tissues and their adjacent normal tissues. We found that IGF2BP2 protein was highly expressed in all three pairs of PAAD tissues than that of normal (Figure 6A and Supplementary Figure 6). Then, 20 pairs of PAAD clinical specimens with their adjacent normal pancreatic tissue were extracted to detect the mRNA expression level of IGF2BP2. It turned out that IGF2BP2 was significantly overexpressed in PAAD cells from clinical samples than normal pancreatic cells (Figure 6B). We further utilized 5 cell lines (four PAAD cell lines: Panc-1, CFPAC-1, BXPC-3, SW1990; one normal pancreatic ductal epithelial cell line: HPDE) to verify IGF2BP2 expression at mRNA and protein level in vitro. qRT-PCR and Western Blotting results further confirmed the presence of up-regulated mRNA and protein expression of IGF2BP2 in PAAD (Figure 6C, 6D).

Apart from the above research, survival analysis was also performed by dividing patients of TCGA_PAAD cohort into two groups according to the median mRNA expression level of every single gene. However, none of the mRNA expression of the 24 genes was found to significantly influence the OS and DFS of PAAD patients (Supplementary Figure 7).

### Predictive potential of *IGF2BP2* for the prognosis of PAAD patients and downstream Enrichment Analysis for *IGF2BP2* overexpression

The results so far made us very interested in the m^6^A “reader” gene *IGF2BP2*. To preliminarily explore the clinical and biological function of IGF2BP2, univariate and multivariate Univariate and multivariate Cox regression analysis was applied to further confirm the relationship between copy number gain of *IGF2BP2* and prognosis of PAAD patients. Results suggested that copy number gain of *IGF2BP2* alone was an independent risk factor for worse OS (for multivariate Cox regression, HR = 2.392, 95%CI: 1.392-4.112, p<0.001) and DFS (for multivariate Cox regression, HR = 2.400, 95%CI: 1.236-4.659, p=0.010) of PAAD patients (Table 4). In summary, we believed that *IGF2BP2* was an important indicator leading to higher severity and poorer prognosis of PAAD patients.

**Table 4:** Univariate and Multivariate COX regression analysis of IGF2BP2 for PAAD patients' overall survival (OS) and disease-free survival (DFS)*.

**Groups**	**OS**				**DFS**			
	**Univariate Cox Regression**		**Multivariate Cox Regression**		**Univariate Cox Regression**		**Multivariate Cox Regression**	
	**HR**	**p**	**HR**	**p**	**HR**	**p**	**HR**	**p**
Age	1.218 (0.780-1.902)	0.386	1.337 (0.838-2.132)	0.223	1.026 (0.641-1.641)	0.915	1.143 (0.698-1.872)	0.596
Gender	0.804 (0.519-1.245)	0.328	0.774 (0.482-1.242)	0.288	0.811 (0.506-1.298)	0.382	0.777 (0.459-1.313)	0.346
M^#^	1.031 (0.666-1.598)	0.89	0.889 (0.553-1.528	0.626	0.701 (0.435-1.129)	0.144	0.660 (0.396-1.102)	0.112
N^#^	**1.799 (1.053-3.073)**	**0.032**	1.212 (0.350-4.250)	0.756	1.395 (0.828-2.351)	0.211	0.928 (0.198-4.347)	0.924
T^#^	0.879 (1.053-3.807)	0.096	1.398 (0.634-3.079)	0.406	1.212 (0.635-2.313)	0.560	1.387 (0.759-2.533)	0.932
Stage	**1.839 (1.048-3.227)**	**0.034**	1.253 (0.332-4.724)	0.739	1.374 (0.808-2.334)	0.241	1.339 (0.282-6.364)	0.714
Grade	1.263 (0.796-2.003)	0.322	1.278 (0.774-4.724)	0.337	1.250 (0.759-2.058)	0.381	1.283 (0.736-2.238)	0.380
Primary Site	1.779 (0.980-3.231)	0.058	1.653 (0.871-3.139)	0.124	1.482 (0.846-2.597)	0.169	1.387 (0.759-2.533)	0.288
Maximum Diameter of resected tumor	1.295 (0.792-2.116)	0.302	1.172 (0.694-1.979)	0.553	1.284 (0.758-2.057)	0.384	1.265 (0.734-2.179)	0.397
**Copy Number Gain of IGF2BP2 or Other Patients**	**2.399 (1.436-4.008)**	**<0.001**	**2.392(1.392-4.112)**	**0.002**	**2.433 (1.317-4.495)**	**0.005**	**2.400 (1.236-4.659)**	**0.010**

^#^TNM: T, primary tumor status; N, lymph node metastasis; M, distant metastasis. *Ambiguous variables (Nx, Mx, N/A, discrepancy and Gx) were excluded.

**Figure 7:**
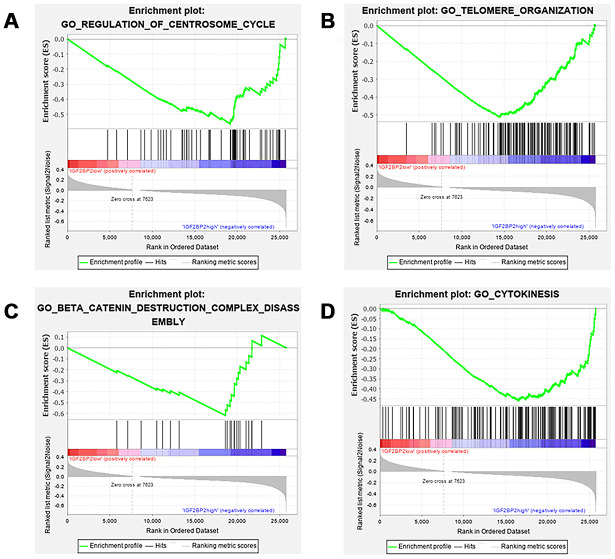
**GSEA results of different expression level of IGF2BP2.** Gene set enrichment plots of (**A**) regulation of centrosome cycle; (**B**) telomere organization; (**C**) β-catenin destruction complex disassembly; (**D**) cytokinesis. Group of IGF2BP2 low expression was compared with group of high expression.

We then determined to explore the role of genetic alteration of *IGF2BP2* in the pathogenesis of PAAD. Gene Set Enrichment Analysis (GSEA) suggested that high expression of *IGF2BP2* was related to some critical biological processes including 1) regulation of centrosome cycle; 2) telomere organization; 3) β-catenin destruction complex disassembly; 4) cytokinesis (Figure 7A–7D and Table 5). All these 4 processes were highly conserved and related to the malignant behavior of tumors, including cell proliferation, cell immortalization and tumor immunization. To preliminarily explore how many m^6^A sites IGF2BP2 could bind to as a m^6^A “reader” protein, RMBase V2.0 (http://rna.sysu.edu.cn/rmbase/index.php) based on epitranscriptome sequencing data was then explored. Result of the data mining showed that IGF2BP2 protein was capable of binding to a total of 60,935 m^6^A sites on tens of thousands of mRNAs. These results might provide some new ideas for subsequent researchers who would like to study the molecular mechanism of IGF2BP2 in pancreatic cancer.

**Table 5:** Gene sets enrichment of high *IGF2BP2* mRNA expression level in PAAD cohort.

**GS DETAILS**	**SIZE**	**ES (absolute value)**	**NES (absolute value)**	**NOM p-val**	**FDR q-val**
GO_REGULATION_OF_CENTROSOME_CYCLE	58	0.56	2.02	<0.001	0.211
GO_TELOMERE_ORGANIZATION	146	0.51	1.99	<0.001	0.143
GO_BETA_CATENIN_DESTRUCTION_COMPLEX_DISASSEMBLY	21	0.62	1.93	0.002	0.228
GO_CYTOKINESIS	154	0.46	1.92	<0.001	0.245

Abbreviations: ES: enrichment score; NES: normalized enrichment score; NOM p-val: normalized p-value; FDR q-val: false discovery rate q value

## DISCUSSIONS

High-throughput sequencing technology related to RNA epitranscriptome has developed rapidly in recent years. M^6^A-Seq, MeRIP-seq and many other innovations gave researchers chances to study transcriptome-wide mapping for m^6^A modification. However, with the explosive growth of sequencing data, how to extract m^6^A -related molecular pathways with biologically significance has become a major challenge facing scientists today. Given this problem, starting with a limited number of m^6^A regulators using bioinformatic methods might be an alternative way to solve it. In this study, we detected a very high frequency of alterations in 24 m^6^A related genes. Furthermore, these alterations are significantly related to the mRNA expression level of m^6^A regulatory genes, implying that the dysregulation of m^6^A regulators might play an important role in PAAD. Mutation events for 24 m^6^A genes occur infrequently in the included patients, with no gene got a mutation frequency of more than 1%. However, we noticed that there was one patient (TCGA ID: TCGA.IB.7651.01) who had mutations in 11 of 24 m^6^A regulatory genes. Limited by sample size and mutation frequency, we could not find more similar patients with large m^6^A mutation burden to conduct statistical studies on them. However, it was possible that the mechanism related to m^6^A gene mutation could explain the onset of this kind of patients which occupied a very small proportion. Digging into the relationship between m^6^A mutations and the pathogenesis of PAAD and designing target therapies about these mutations might have the potential to benefit these patients. High frequency of CNA events has been previously reported in similar researches relating to clear cell renal cell carcinoma (ccRCC) [17] and acute myelocytic leukemia (AML)[18]. However, as shown in our research, CNA patterns in PAAD were totally different from other malignancies that was previously reported. In ccRCC or AML patients, most observed CNA events in writer genes resulted in copy number loss with down-regulation of the corresponding mRNAs, while CNAs of the eraser genes mainly led to overexpression and gain of function. However, in our research, copy number loss of both writers and erasers dominated most of the CNA events, especially in *WTAP*, *METTL16* and *ALKBH5*. We suggested that these alterations might be related to the inhibition of m^6^A modification and the decrease of m^6^A sites at global level. Further research is urged to verify our hypothesis and to interpret the specific biological significance behind it in PAAD. Besides, owing to the discovery of many new reader proteins in the past few years, our study had the most “reader” genes included compared with other similar m^6^A studies. CNA patterns of the reader genes showed an interesting trend in our research as well. Specifically, YTHDC and YTHDF family which promoted mRNA degradation [19] exhibited a tendency of copy number loss and down-expression of mRNAs, but genes from IGF2BP family which functioned as mRNA stabilizers [20] were dominated by the pattern of copy number increase and gain of function. This tendency implied us that there might be global improvements in mRNA stability at the whole transcriptome level of PAAD, and m^6^A modification, especially m^6^A “reader” proteins, might play a pivotal role in this mechanism. This result might yield a high value for further research.

Linking genomic and transcriptomic alterations to patients' clinicopathological manifestations and prognostic values has been an obvious trend in bioinformatic analysis over the past years. This interdisciplinary research combines the advantages of both clinicians and bioinformatic experts, showing great value of transforming medicine. In this study, we also evaluated the effect of m^6^A gene alterations on clinicopathological factors and survival of PAAD patients. It turned out that patients with CNAs or mutations of m^6^A regulatory genes were significantly corelated with the patient being male, tumor diameter greater than 3 cm, and advanced pathological grade. These results further demonstrated that changes in m^6^A regulatory genes were positively correlated with the increased clinical malignancy of PAAD. However, results of survival analysis were slightly different from what we expected and what other research reported. Genes with high CNA frequency did not exhibit significant relationship with the overall and disease-free survival. The only gene among the 24 regulators associated with OS and DFS was the “reader” gene *IGF2BP2*. Patients who over expressed IGF2BP2 had a significantly poorer prognosis. The subsequent Cox regression analysis further proved that *IGF2BP2* gain of number is an independent predictor of OS and DFS in PAAD patients. This result was in line with Hu X and his colleagues’ research [21] that *IGF2BP2* “gain of function” was positively correlated with the poorer prognosis of PAAD patients. Besides, worse OS in patients with copy number gain of “reader” gene cluster in combination with writer or eraser gene loss was observed, making it clearer that m^6^A reader genes played a more important role in PAAD patients. Given that there are far fewer studies focusing on m^6^A readers than those on writers and erasers like *METTL3*, *WTAP* and *FTO*, we believed that these results could guide the subsequent research on m^6^A in PAAD towards the biological function of “reader” proteins and their molecular mechanism. We had to admit that the limited case number and the partially missing data of the included datasets in our study restricted the accuracy of our results to some extent. Detecting the m^6^A level directly through m^6^A-seq and evaluating its effect on PAAD survival in a new cohort with more patients and complete information are now urged for more statistically significant results. Another potentially controversy may be our differential expression analysis with RNA-seq data, as none of the 24 m^6^A genes has met the conventional standard of differential expression (usually log2FoldChange > 1.5, FDR < 0.05). It is necessary for us to explain that due to the utilization of multiple datasets from different sources, we used a more rigorous solution to remove batch effects, which to a large extent made us underestimate the degree of mRNA differential expression.

*IGF2BP2* was an unexpected gain in our study. Insulin-like growth factor 2 mRNA binding protein (IGF2BP) family is a class of single-stranded RNA binding proteins (RBPs) that are highly conserved in eukaryotes. The common feature of the three members in this protein family is that they consist of 6 domains, including 2 RNA recognition domains and 4 K-H motifs [22]. IGF2BP family had not been confirmed to function in m^6^A modification until 2018, when Huang H [20] found that IGF2BP family could recognize m^6^A modified “GG (m^6^A) C” sequence that was highly conserved in mammals. Moreover, the combination between IGF2BP family and target mRNAs enhanced mRNA stability and promoted subsequent translation processes, resulting in increased expression level of target proteins; IGF2BP proteins could also guide ribosome-bound mRNAs (ie, ribonucleoprotein complex) into stress granules to ensure stable storage of mRNAs under stress condition. Since then, there have been a few more studies exploring IGF2BP family as m^6^A reader to activite tumorigenesis and cancer development. Huang X et al [23] reviewed in 2018 that *IGF2BP1* was over-expressed in diverse types of cancers, including lung cancer, liver cancer, leukemia and many other diseases; this up-regulation showed cancer-promoting effect by stabilizing many mRNAs and non-coding RNAs, like KRAS, MYC, let-7 et al. Similarly, *IGF2BP3* [24] was also highly expressed in a variety of cancers, promoting carcinogenesis through its RBP function. However, no one has published research so far to prove their expression and function in PAAD. Although they have a relatively high incidence of mutations and CNA events in PAAD, results did not support their association with the prognosis and clinicopathological factors of PAAD patients as well. In pancreatic cancer, Hu X [21] found that IGF2BP2 recognized and bound to long non-coding RNA DANCR (lncDANCR), which increased the stability of DANCR and promoted the development of PAAD. Li T et al [25] confirmed that IGF2BP2 could recognize and bind with the m^6^A enriched site of the CDS region on *SOX2* mRNA and further inhibited degradation of *SOX2* to promote the occurrence and development of colorectal cancer. GSEA analysis in our research for the pathways affected by IGF2BP2 overexpression in PAAD suggested that high expression of *IGF2BP2* was related to the following biological processes: 1) regulation of centrosome cycle; 2) telomere organization; 3) β-catenin destruction complex disassembly; 4) cytokinesis. These pathways were closely related to the pathogenesis of cancer in different ways. They had close associations with a variety of malignant biological behaviors such as abnormal mitosis, immortalization of tumor cells, epithelial-mesenchymal transition, tumor invasion and migration, and tumor immune surveillance and immune escape [26–29]. These results provided preliminary clues for those who would like to study the mechanism of IGF2BP2 in PAAD. Supported by search results from database RMBase, IGF2BP2 was also capable to target tens of thousands of mRNAs because of its affinity with the highly conservative "GG(m^6^A)C" motif. We suggested that meRIP-seq might be applied to screen all the significant targets in PAAD and to further reveal the molecular mechanism of IGF2BP2.

In conclusion, this study explored the alterations of m^6^A regulators from the perspective of genomics, transcriptomics, and clinical information in PAAD for the first time by utilizing TCGA dataset of PAAD and multiple bioinformatic methods. An obvious correlation was found between the alterations of m^6^A regulatory genes and worse clinical characteristics including survival. A m^6^A reader protein, IGF2BP2, was of great importance in m^6^A modification of PAAD. This study has established a bridge between the microscopic RNA modification mechanism of m^6^A and the macroscopic clinical manifestations of PAAD patients, highlighting the value of m^6^A regulatory genes, especially *IGF2BP2*, in the pathogenesis, diagnosis and treatment of PAAD. To further confirm our results and clarify the definite target mRNA of the m^6^A modification during PAAD initiation and progression, studies in another larger PAAD cohort with m^6^A -Seq and MeRIP-seq will be helpful.

## MATERIALS AND METHODS

### Ethics statement

All the CNAs, mutations, mRNA expression data and clinical information were retrieved from TCGA database by cBioportal platform [30] through R package “cgdsr” and UCSC-Xena program [31] which are open sourced to the public under certain guidelines. The ICGC database was further applied to prove TCGA results. Thus, it is confirmed that all written informed consent was achieved. For patients’ tissue array from the third Xiangya hospital, written informed consent was obtained from all patients and the study was approved by the ethics committee of the third Xiangya Hospital.

### Data processing

We identified 3 datasets (TCGA_PAAD, ICGC_AU_PAAD and ICGC_CA_PAAD) with complete clinical information, of which all three datasets included somatic mutation and RNA-seq raw data, while CNA data was only available in TCGA_PAAD cohort. For somatic mutation research, raw file in “MAF” format were got from R package “TCGAmutations” and ICGC data portal. The R packages “Maftools” and “Genvisr” were then applied to analyze mutation frequency and draw “waterfall plot”. For CNA research, the loss and gain of copy number changes was identified using segmentation analysis and GISTIC algorithm. When clinical significance of the status of CNA and/or mutation was discussed, this PAAD cohort was usually divided into two subgroups, “with CNAs and/or mutation of all m^6^A regulatory genes (or a certain m^6^A regulatory gene)” and “without CNA and mutation”. The mRNA expression data were calculated from RNA-Seq V2 RSEM release and log2 fold change was applied before analyzing the relationship between CNA and mRNA expression. The R package “edgeR” [32] was utilized to analyze mRNA differential expression.

### Statistical analysis

All statistical data and figures were analyzed by R (Lucent Technologies, New Jersey, USA) or GraphPad Prism 8.0 (GraphPad Software, La Jolla, CA, USA) to ensure the aesthetics and editability of the figures and tables. The association between CNAs of m^6^A regulatory genes and clinicopathological characteristics were analyzed using chi-square test or Mann-Whitney U test. Kaplan-Meier curve and log-rank test were used to evaluate the prognosis value of all kinds of CNAs that the frequency is greater than 15% of total sample number. Cox proportional hazard regression model was performed using R package “survival” [33] and “survminer” [34]. All statistical results with a p-value <0.05 were considered significant.

### Clinical samples

A total of 20 pancreatic cancer tissues and adjacent normal tissues were collected from Department of Hepatobiliary and Pancreatic Surgery, The Third Xiangya Hospital. All tissues were frozen immediately in liquid nitrogen after surgical excision and stored at −80°C. Clinicopathological information was retrieved from the hospital database. The ethic approval was mentioned above.

### Quantitative real-time PCR (qRT-PCR), Western Blotting (WB) and Immunohistochemistry (IHC)

For qRT-PCR, Total RNAs were isolated from PC tissues and cell lines by TRIzol reagent (Invitrogen) and reverse transcription was performed with a Prime Script RT reagent kit (Takara Biotechnology, China). Qrt-PCR reactions were carried out on Applied Biosystems 7500 Real-time PCR Systems (Thermo Fisher Scientific). GAPDH was used as control for IGF2BP2 mRNA expression. The relative expression level of the target RNA was calculated by 2^−ΔΔCt^. In our study, all involved primers were produced by Sangon Biotech (Shanghai, China).

For WB analysis, Cells were lysed in 1x RIPA buffer with 1mM PMSF and 1x complete protease inhibitor cocktail (Roche, Basel, Switzerland). Western blottings were conducted under standard procedures. Briefly, Protein lysates were separated by SDS-PAGE, and electrophoretically transferred to PVDF membrane from MilliporeTM with nominal pore size of 0.22 um. After blocking, the membrane was incubated at 4°C overnight with anti-IGF2BP2 (Proteintech) or β-Actin (Santa Cruz Biotechnology) diluted in non-fat dry-milk (Santa Cruz Biotechnology), followed by HRP-labeled secondary (Santa Cruz Biotechnology) antibody incubation. Chemiluminescence signal was developed by ECL Plus Western Blotting Detection Reagents (GE Healthcare Life Sciences, Munich, Germany).

For IHC test, IHC staining of IGF2BP2 was conducted on tissue microarray consisting of 12 PDAC tissues and 12 paired normal tissues. IHC staining was carried out using Histostain-Plus kit according to manufacturer’s protocol. Briefly, antigen retrieval was conducted by heating the sections in boiling sodium citrate buffer for 20 mins. After 3% hydrogen peroxide and BSA blocking, the tissues were incubated with anti-IGF2BP2 (Proteintech, Chicago, IL) at 4°C overnight. After washing, the tissues were incubated with HRP-conjugated secondary antibody (Santa Cruz Biotechnology, Santa Cruz, CA). IHC signal was developed by DAB substrate, and counter-stained by haematoxylin. Three random fields at 200x magnification were captured per sections for evaluation. Scoring of the IHC was based on the percentage of positive cells and staining intensity under a light microscope. Four categories (0, 1+, 2+ and 3+) were denoted as negative, weak, moderate and strong respectively, and final score was calculated by averaging the score of three fields.

### Downstream pathway and target analysis for IGF2BP2

Gene set enrichment analysis (GSEA) provided by the JAVA program (Version 4.0.3) with MSigDB v6.1 was applied to explore the downstream biological processes affected by differential expression of IGF2BP2. Patients in the cohort was divided into two groups according to the median expression level of IGF2BP2. Finally, 25880 genes were enrolled into the GSEA process. Hallmark gene set “c5.bp.v7.0.symbols.gmt” was used in this study [35]. Gene sets which got highest Enrichment Score (ES) with normalized p-value <0.05, and the false discovery rate (FDR) <0.25 were considered significantly enriched.

## Supplementary Material

Supplementary Figures

Supplementary Table 1

Supplementary Table 2
