# Supplementary material for: Multiomics integrative analysis for gene signatures and prognostic values of m6A regulators in pancreatic adenocarcinoma: a retrospective study in The Cancer Genome Atlas project
Source: Aging (Albany NY). 2020 Oct 20;12(20):20587–610. doi: 10.18632/aging.103942 (PMC7655159; doi:10.18632/aging.103942)
Supplement: Supplementary Figures [file aging-12-103942-s001..docx]

SUPPLEMENTARY FIGURES


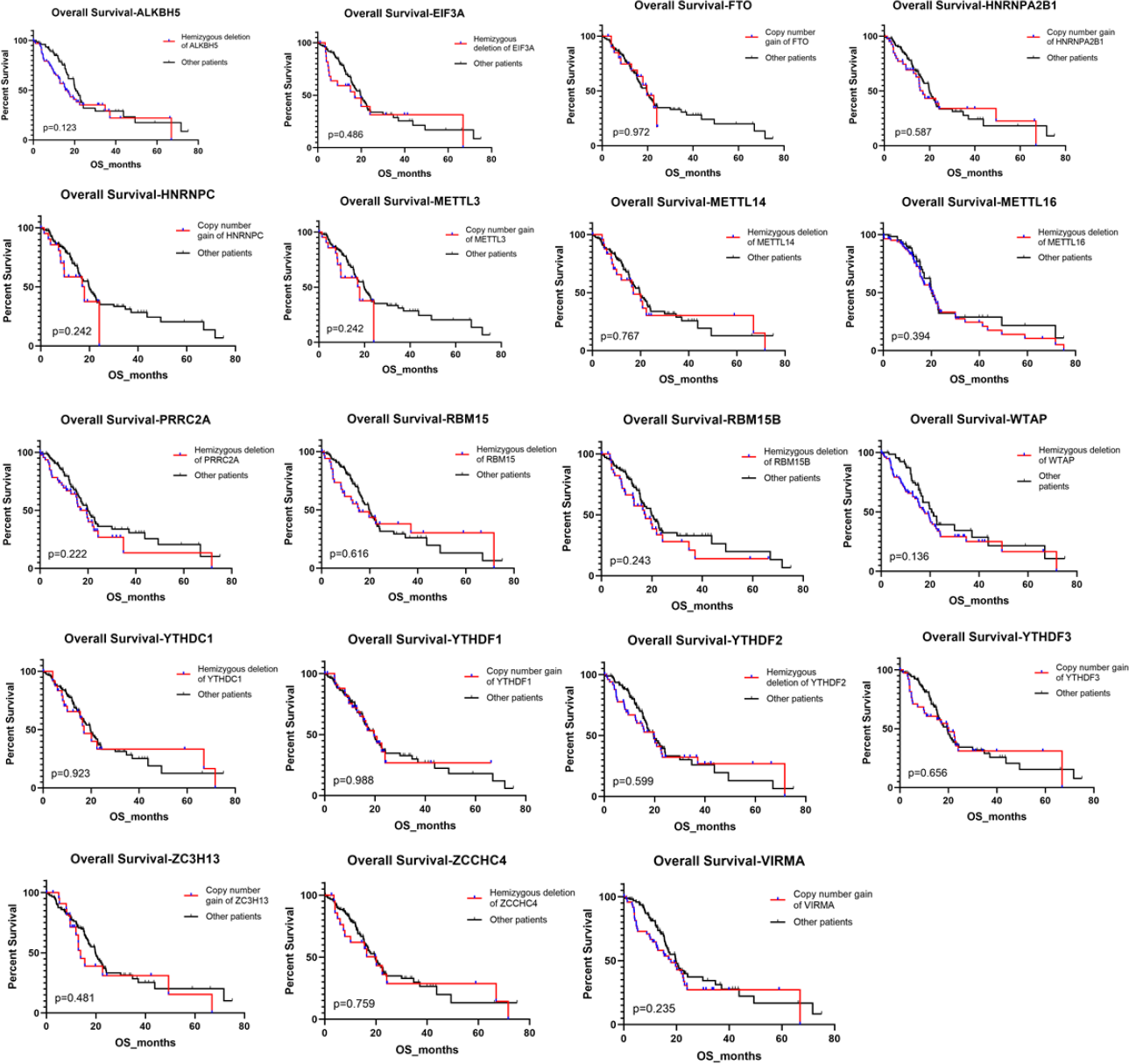


Supplementary Figure 1. Overall survival (OS) of PAAD patients with different kinds of CNA of m^6^A regulatory genes. The results of all survival analysis showed no statistical significance (p≥0.05), except for IGF2BP2 in Figure 3C.


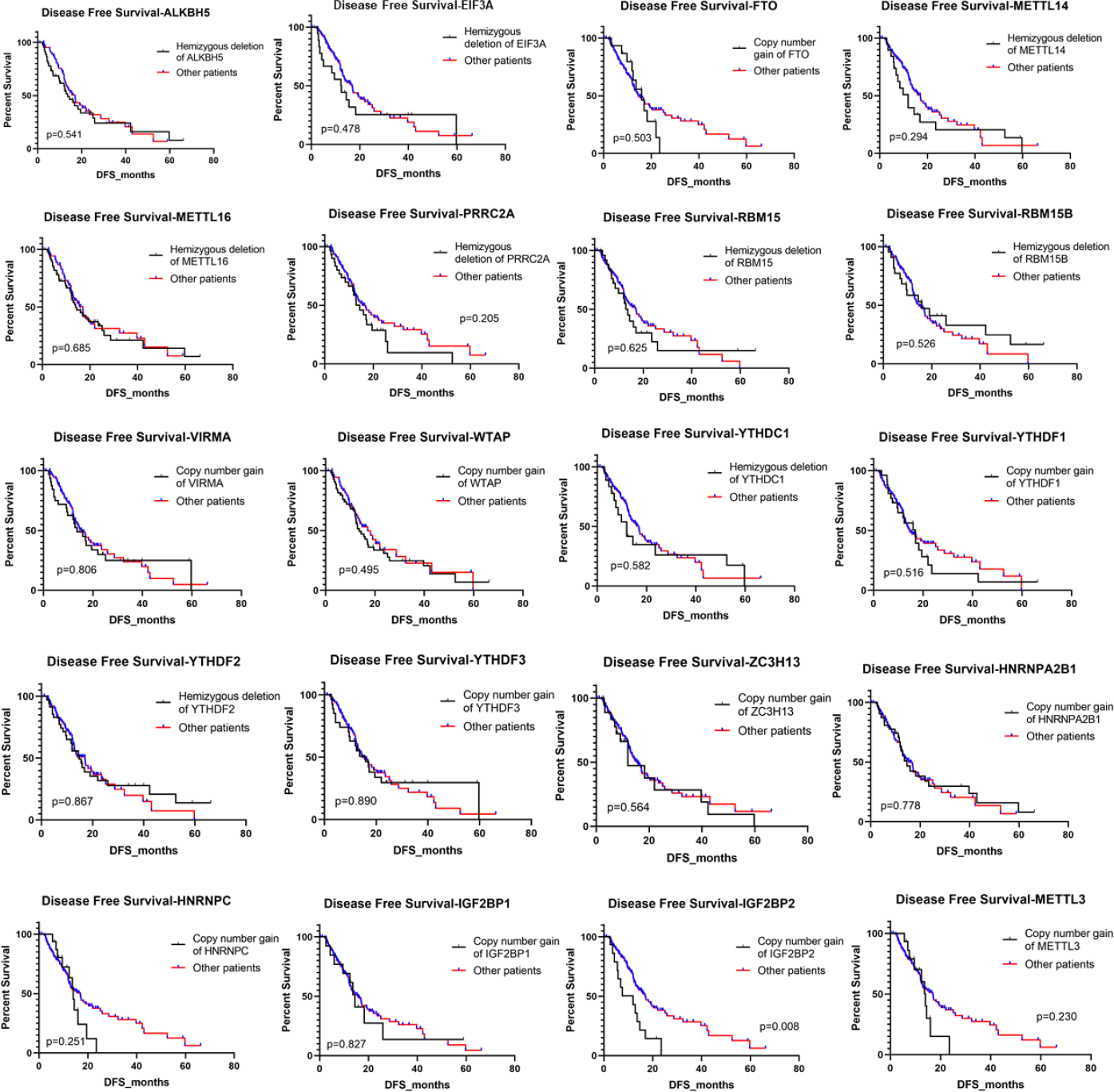


Supplementary Figure 2. Disease-free survival (DFS) of PAAD patients with different kinds of CNA of m^6^A regulatory genes. The results of all these survival analysis showed no statistical significance (p≥0.05), except for IGF2BP2 in Figure 3C.


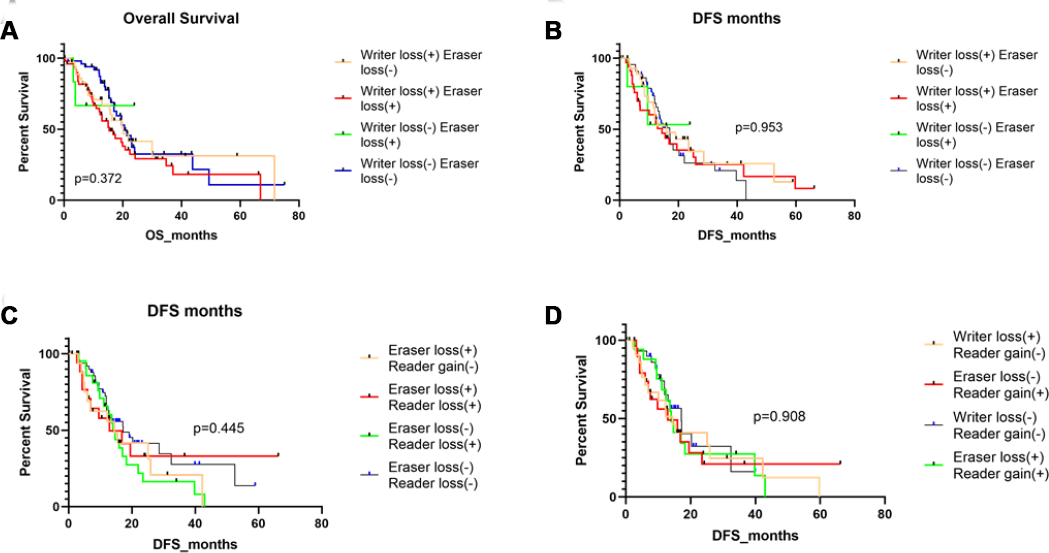


Supplementary Figure 3. OS and DFS of PAAD patients with simultaneous alterations of writers, erasers and readers genes. The results of all the survival analysis showed no statistical significance (p≥0.05). (A) OS of PAAD patients with simultaneous alterations of writer genes and eraser genes. (B) DFS of PAAD patients with simultaneous alterations of writer genes and eraser genes. (C) DFS of PAAD patients with simultaneous alterations of eraser genes and reader genes. (D) DFS of PAAD patients with simultaneous alterations of writer genes and reader genes. The results of all the above survival analysis showed no statistical significance (p≥0.05).


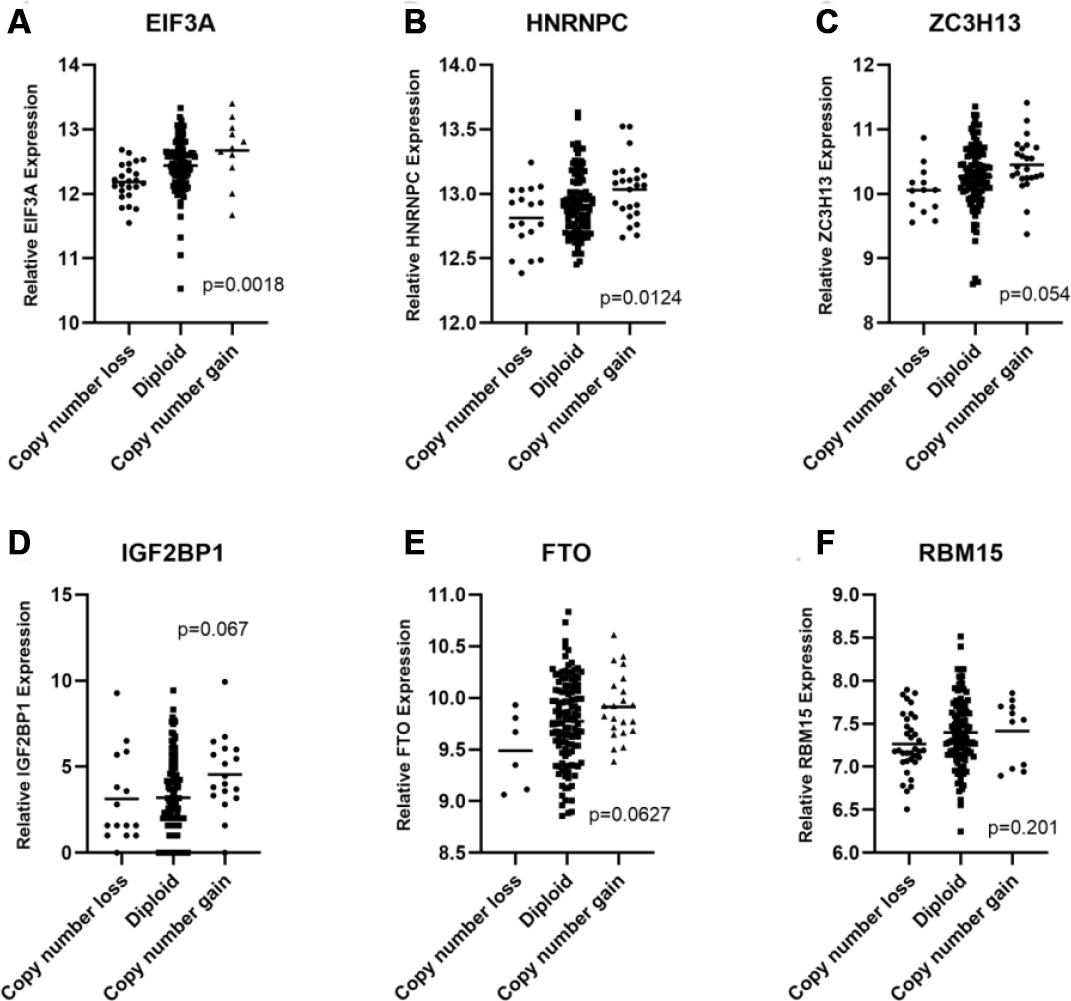


Supplementary Figure 4. Correlation between different CNA patterns and mRNA expression levels of m^6^A regulatory genes. Only when p≥0.001 the gene was included in these figures shown in (A–C), EIF3A, HNRNPC and ZC3H13 showed significant correlation between their CNAs and mRNA expression. However, in (D–F), IGF2BP1, FTO and RBM15 showed no significant correlation between their CNAs and mRNA expression (p≥0.05).


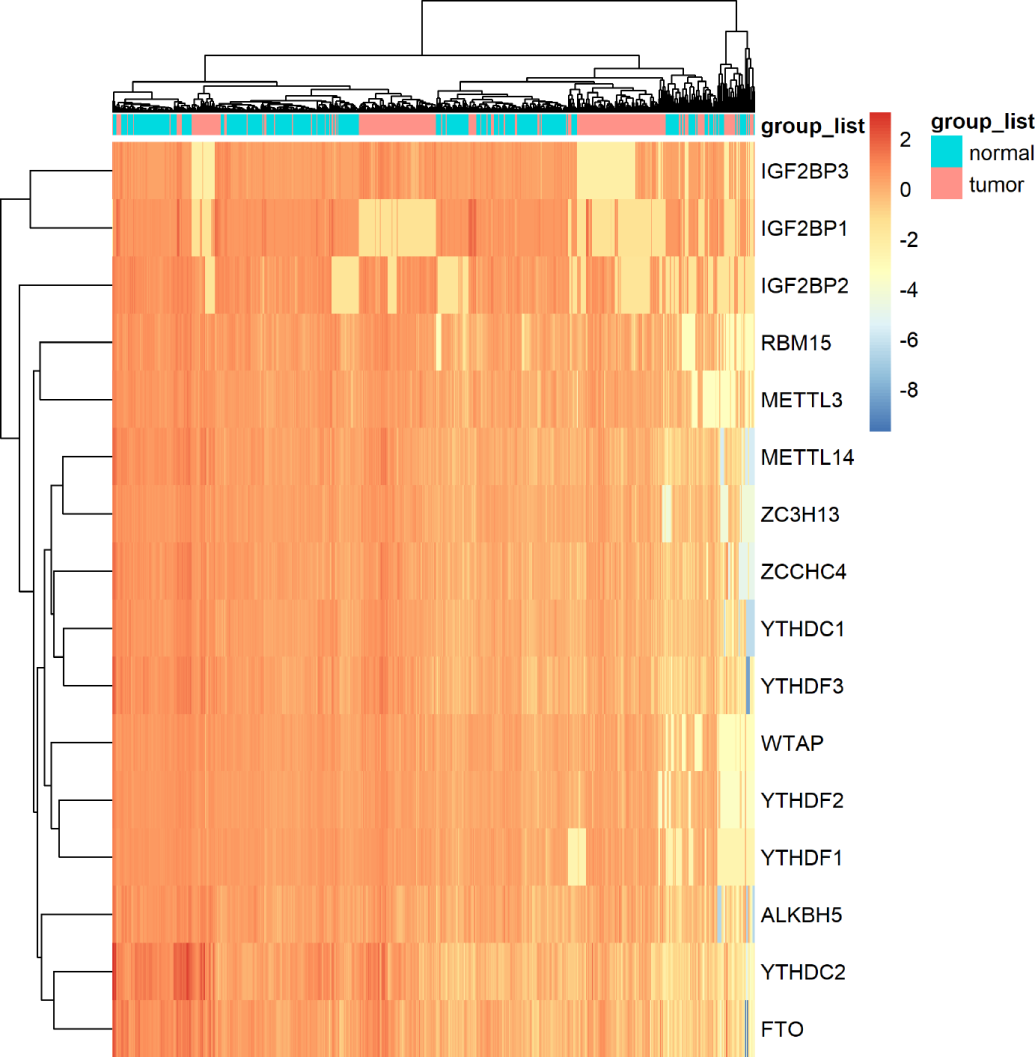


Supplementary Figure 5. mRNA expression heatmap for all m^6^A regulatory genes. RNA-sequencing data was from TCGA_PAAD project and GTEx database. Batch effect was eliminated before differential expression analysis was done.


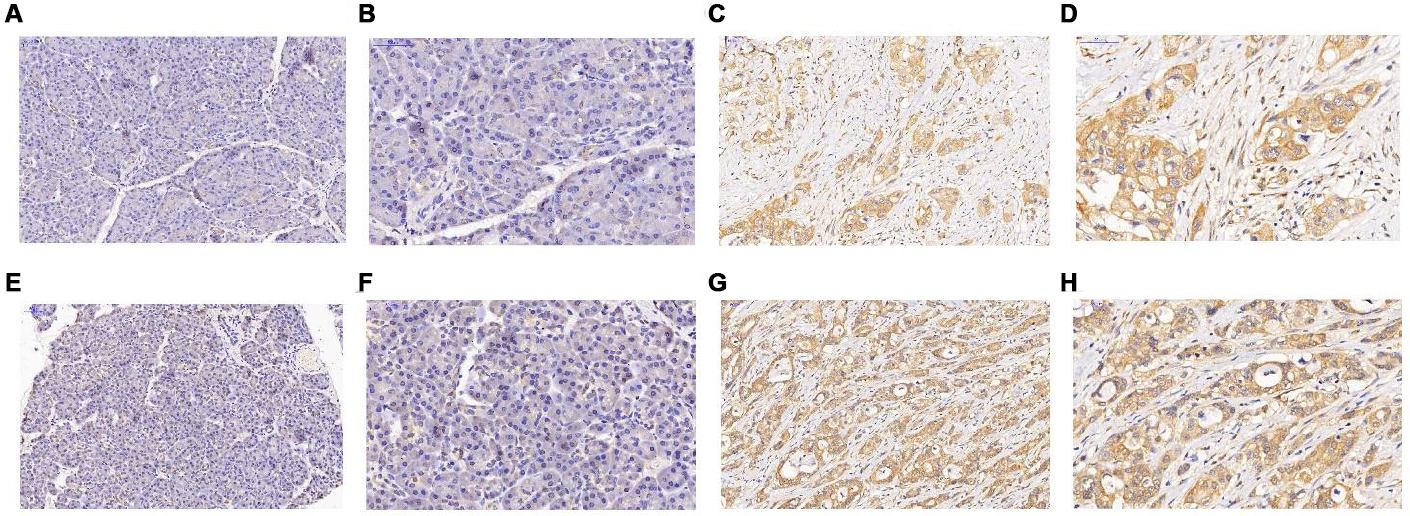


Supplementary Figure 6. Immunohistochemistry staining (IHC) for another 2 pair of PAAD tissues and their adjacent normal tissues for IGF2BP2 protein. (A–D) for patient NO.2 and (E–H) for patient NO.3. (A, B, E, F) showed normal pancreas tissues and (C, D, G, H) showed the corresponding PAAD tissue staining. (A, C, E, G) were magnified 200 times (200x), and for (B, D, F, H), 400 times (400x).


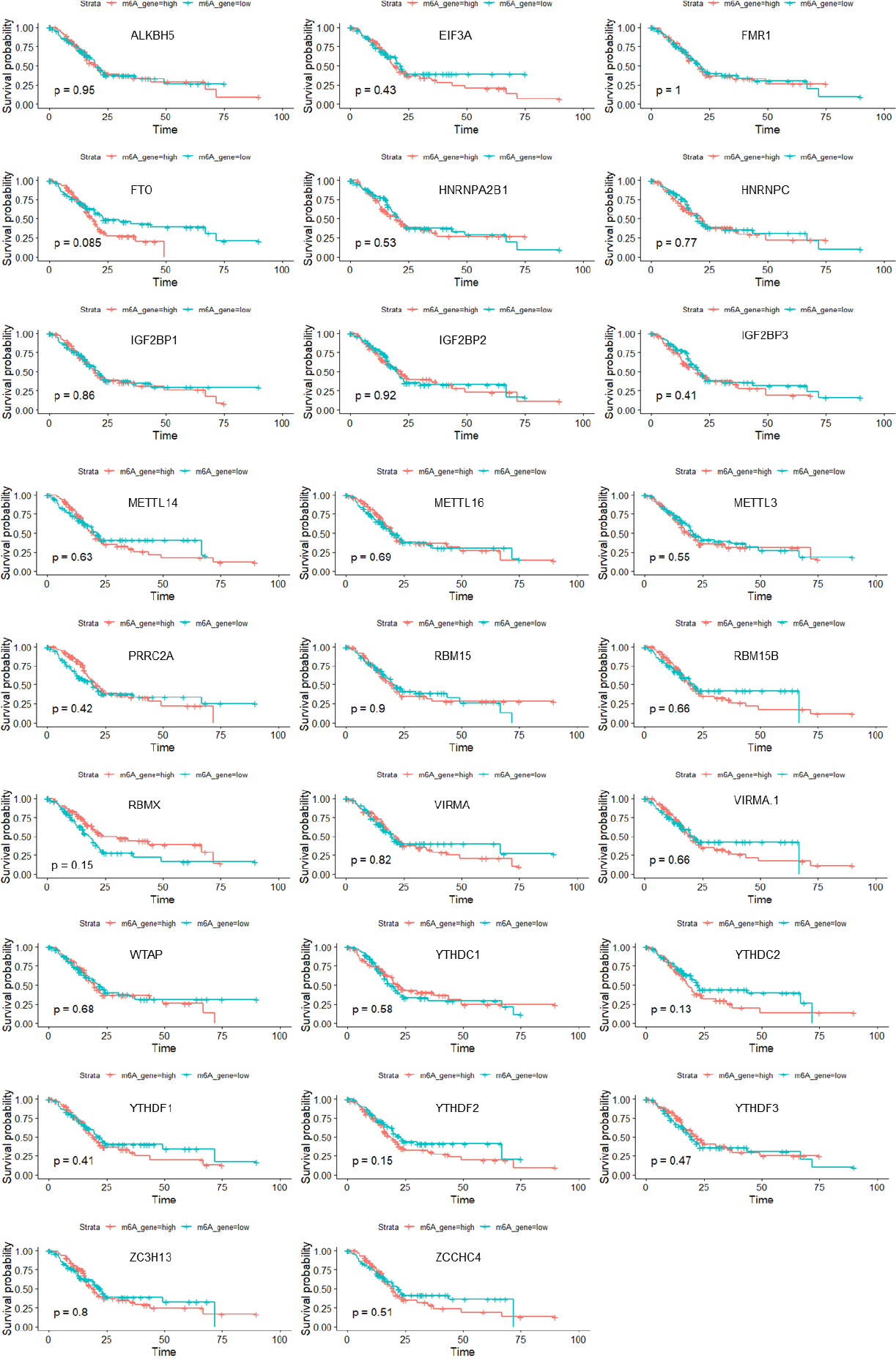


Supplementary Figure 7. Overall survival (OS) of PAAD patients with high or low expression of m^6^A regulatory genes. Definition of “high” and “low” expression groups were based on the median level of every single mRNA. No statistically significant results were found in the analysis.
