# Supplementary material for: Multiomics integrative analysis for gene signatures and prognostic values of m6A regulators in pancreatic adenocarcinoma: a retrospective study in The Cancer Genome Atlas project
Source: Aging (Albany NY). 2020 Oct 20;12(20):20587–610. doi: 10.18632/aging.103942 (PMC7655159; doi:10.18632/aging.103942)
Supplement: Supplementary Table 1 [file aging-12-103942-s002..docx]

**Supplementary Table 1. Mutations of m^6^A regulatory genes in 616 ICGC_PAAD patients.**

| **ICGC_PAAD Sample ID** | **IGF2BP3** | **METTL14** | **FMR1** | **YTHDC1** | **FTO** | **WTAP** | **IGF2BP2** | **ZCCHC4** | **ALKBH5** | **ZC3H13** | **YTHDF1** | **PRRC2A** | **EIF3A** | **RBM15** | **RBM15B** | **IGF2BP1** | **YTHDF3** | **YTHDC2** |
| --- | --- | --- | --- | --- | --- | --- | --- | --- | --- | --- | --- | --- | --- | --- | --- | --- | --- | --- |
| MU28793631 |  |  |  |  |  |  |  |  |  |  |  |  |  | V459G |  |  |  |  |
| MU12476718 |  | Y119C, Y81C |  |  |  |  |  |  |  |  |  |  |  |  |  |  |  |  |
| MU155394 |  |  |  |  |  | R162Q |  |  |  |  |  |  |  |  |  |  |  |  |
| MU1677389 |  |  |  |  |  |  |  |  |  | R306* |  |  |  |  |  |  |  |  |
| MU1678398 |  |  | R500W, R512W, R533W, R508W, R180W, R510W |  |  |  |  |  |  |  |  |  |  |  |  |  |  |  |
| MU1679195 |  |  |  |  |  |  |  |  |  |  |  | A565V |  |  |  |  |  |  |
| MU1679568 |  |  |  |  |  |  |  |  |  |  |  | E32K |  |  |  |  |  |  |
| MU1679586 |  |  |  |  |  |  |  |  |  |  |  | R213H |  |  |  |  |  |  |
| MU1679599 |  |  |  |  |  |  |  |  |  |  |  | W423S |  |  |  |  |  |  |
| MU1679614 |  |  |  |  |  |  |  |  |  |  |  | E443K |  |  |  |  |  |  |
| MU1679634 |  |  |  |  |  |  |  |  |  |  |  | D1064N |  |  |  |  |  |  |
| MU1682004 |  |  |  |  |  |  |  |  |  |  |  |  |  |  |  |  |  | R953Q |
| MU1684657 |  | R260H, R298H |  |  |  |  |  |  |  |  |  |  |  |  |  |  |  |  |
| MU1685683 |  |  |  |  |  |  |  |  |  |  | G409S, G459S |  |  |  |  |  |  |  |
| MU1686594 |  |  | E595D, E574D, E576D, R526I, E566D, E616D, E246D |  |  |  |  |  |  |  |  |  |  |  |  |  |  |  |
| MU1688142 |  |  |  |  |  |  |  |  |  |  |  |  |  |  |  |  |  | K1294M |
| MU1690481 | I503T |  |  |  |  |  |  |  |  |  |  |  |  |  |  |  |  |  |
| MU1690943 | R233H |  |  |  |  |  |  |  |  |  |  |  |  |  |  |  |  |  |
| MU1694757 |  |  |  |  |  |  |  |  |  |  |  |  |  |  |  |  |  | L1074F |
| MU1695483 |  |  | R190Q |  |  |  |  |  |  |  |  |  |  |  |  |  |  |  |
| MU1861952 |  |  |  |  |  |  |  |  |  | R784* |  |  |  |  |  |  |  |  |
| MU20990916 |  |  |  |  |  |  |  |  |  |  |  |  |  | A939E |  |  |  |  |
| MU23349411 |  |  |  |  |  |  |  |  |  |  |  |  | A289V, A255V |  |  |  |  |  |
| MU28660142 | I54V |  |  |  |  |  |  |  |  |  |  |  |  |  |  |  |  |  |
| MU30276402 |  | R298C, R260C |  |  |  |  |  |  |  |  |  |  |  |  |  |  |  |  |
| MU30646279 |  |  |  |  |  |  |  |  |  |  |  |  |  |  |  |  | R446H, R257H |  |
| MU30892194 |  |  |  |  |  |  | I457M, I520M, I514M, I471M |  |  |  |  |  |  |  |  |  |  |  |
| MU31084204 |  |  |  |  |  |  |  |  | R52W, R393W |  |  |  |  |  |  |  |  |  |
| MU31216707 |  |  |  |  |  |  |  | P365L, P229L |  |  |  |  |  |  |  |  |  |  |
| MU32108372 |  |  |  |  |  |  |  |  |  |  |  |  |  |  |  | I428M, I289M |  |  |
| MU3882877 |  |  |  |  | V94I, V493I, V197I, V115I |  |  |  |  |  |  |  |  |  |  |  |  |  |
| MU3883055 |  |  |  |  |  |  |  |  |  |  |  |  |  |  |  | L181I, L320I |  |  |
| MU3884301 |  |  |  |  |  |  | R323K, R266K, R329K |  |  |  |  |  |  |  |  |  |  |  |
| MU3884662 |  |  |  |  |  |  |  |  |  |  |  |  |  |  |  | E445K, E306K |  |  |
| MU43628009 |  |  |  |  |  |  |  |  |  |  |  |  |  |  | Y517C |  |  |  |
| MU6301722 |  |  |  |  |  | A159V |  |  |  |  |  |  |  |  |  |  |  |  |
| MU63679993 |  |  |  |  |  |  |  |  |  |  |  |  |  | G297D |  |  |  |  |
| MU63682 |  |  |  |  |  |  |  |  |  | R120*, R1164* |  |  |  |  |  |  |  |  |
| MU68589713 |  |  |  | R4*, R563*, R581*, R589* |  |  |  |  |  |  |  |  |  |  |  |  |  |  |
| MU69648709 |  |  |  |  |  |  |  |  |  | R595R, R1639R |  |  |  |  |  |  |  |  |
| MU75351225 |  |  |  |  |  |  |  |  |  |  |  | R1223H |  |  |  |  |  |  |
| MU8233441 |  |  |  |  |  |  |  |  |  |  |  |  |  |  |  |  | Q131*, Q320* |  |
| MU8978637 |  |  | N608I, N579I, N589I, N259I, N587I, N629I |  |  |  |  |  |  |  |  |  |  |  |  |  |  |  |
| MU9193766 |  |  |  | R568Q, R550Q |  |  |  |  |  |  |  |  |  |  |  |  |  |  |
| MU92794060 |  |  |  |  |  |  |  |  |  |  |  | S1219P |  |  |  |  |  |  |
| MU92982445 |  |  |  |  |  |  |  |  |  |  |  | R1050Q |  |  |  |  |  |  |
| MU94144379 |  |  |  |  |  |  |  |  |  |  |  |  | M1083L, M1049L |  |  |  |  |  |
| MU94194476 |  |  |  |  |  |  |  |  |  |  |  |  |  |  |  |  | L177F |  |
| MU94455611 |  |  |  |  |  |  |  |  |  |  |  | G302V |  |  |  |  |  |  |
| MU94620853 |  |  |  |  |  | W145G |  |  |  |  |  |  |  |  |  |  |  |  |
| MU95644 |  |  |  |  | R322*, R26* |  |  |  |  |  |  |  |  |  |  |  |  |  |
