# Supplementary material for: Multiomics integrative analysis for gene signatures and prognostic values of m6A regulators in pancreatic adenocarcinoma: a retrospective study in The Cancer Genome Atlas project
Source: Aging (Albany NY). 2020 Oct 20;12(20):20587–610. doi: 10.18632/aging.103942 (PMC7655159; doi:10.18632/aging.103942)
Supplement: Supplementary Table 2 [file aging-12-103942-s003..pdf]

SUPPLEMENTARY TABLE

Supplementary Table 2. Differential expression status of m6A regulatory genes in TCGA_PAAD patients compared with GTEx normal individuals.

	log2FoldChange*	p-value
YTHDC2	-0.099	0.012
METTL14	-0.120	0.024
YTHDC1	-0.113	0.045
IGF2BP2	0.341	0.048
ZCCHC4	0.103	0.078
IGF2BP3	0.166	0.389
FTO	0.038	0.394
YTHDF2	-0.090	0.403
YTHDF3	-0.031	0.479
ZC3H13	0.063	0.483
RBM15	0.064	0.574
YTHDF1	-0.062	0.672
WTAP	-0.036	0.774
METTL3	-0.027	0.816
ALKBH5	-0.012	0.861
IGF2BP1	0.040	0.876
YTHDC2	-0.099	0.012
METTL14	-0.120	0.024

*log2FoldChange < 0 meant that a certain gene was down-expressed in PAAD tissue than that in normal.
